# Rainfall erosivity index for the Ghana Atomic Energy Commission site

**DOI:** 10.1186/s40064-016-2100-1

**Published:** 2016-04-15

**Authors:** Paul Essel, Eric T. Glover, Serwaa Yeboah, Yaw Adjei-Kyereme, Israel Nutifafa Doyi Yawo, Mawutoli Nyarku, Godfred S. Asumadu-Sakyi, Gustav Kudjoe Gbeddy, Yvette Agyiriba Agyiri, Evans Mawuli Ameho, Emmanuel Atule Aberikae

**Affiliations:** National Radioactive Waste Management Centre, NNRI, GAEC, P.O. Box LG 80, Accra, Ghana

## Abstract

Rainfall erosivity is the potential ability for rainfall to cause soil loss. The purpose of this study was to estimate the rainfall erosivity index for the Ghana Atomic Energy Commission site in order to compute the surface erosion rate. Monthly rainfall data, for the period 2003–2012 were used to compute annual rainfall erosivity indices for the site, using the Modified Fournier index. Values of the annual rainfall erosivity indices ranged from 73.5 mm for 2004 to 200.4 mm for the year 2003 with a mean annual erosivity index of 129.8 mm for the period. The Pearson’s Coefficient of Correlation was used to establish the relationship between annual rainfall and annual rainfall erosivity. This showed a high degree of positive relationship (r = 0.7) for the study area. The computed mean annual erosivity index revealed that the site is in the high erosion risk zone. Therefore, it is necessary to develop soil protection and management strategies to protect the soil from erosion.

## Background

Rainfall is a key contributing factor to land degradation such as soil erosion. This is as a result of the ability of rainfall to dissolve, loosen or worn away soil by the force of raindrops, runoffs, and river flooding and deposit in other places (Balogun et al. 2012; World Meteorological Organization 2005). Generalized maps of the geographical distribution of rainfall and wind erosion, positions Ghana in the area predominantly vulnerable to rainfall erosion. The demand for land and agricultural products due to population growth is likely to aggravate the problem (Oduro-Afriyie 1995; Norman 1981).

The rate at which the soil at the Ghana Atomic Energy Commission (GAEC) site is eroded is of much concern as some of the nuclear installations of the Commission are underground and requires the soil to be conserved. Ghana is implementing the Borehole Disposal Concept (BDC), a specially engineered borehole, 30–100 m deep with narrow diameter (0.26 m) designed to dispose disused radioactive sources of less than 110 mm in length and 15 mm in diameter. This concept was developed in South Africa under the International Atomic Energy Agency’s (IAEA) AFRA project for the disposal of disused sealed radioactive sources (DSRS) in member states with relatively small DSRS inventories.

To perform the Post Closure Safety Assessment on the proposed Borehole Disposal Facility (BDF) for Ghana, one of the key parameters that needs to be investigated at the proposed site is the surface erosion rate. This parameter will enable us ascertain the duration for the closure zone (the zone between the disposal zone and the ground surface) of the BDF to be eroded for the disposed waste to be uncovered. To be able to compute the surface erosion rate, the rainfall erosivity index for the site is prerequisite hence the need to compute this parameter.

Rainfall erosivity is a function of its amount, duration, drop size and drop size distribution, terminal velocity, intensity and kinetic energy. The significance of rainfall erosivity in the assessment of soil erosion risks stems from the fact that, unlike other natural factors that affect soil erosion, the erosive capacity of rainfall is not subject to human modification (Balogun et al. 2012; Anugulo-Martinez and Begueria 2009; Salako 2003).

Rainfall has an erosive force that is expressed as rainfall erosivity. Rainfall erosivity ruminates the rainfall amount and intensity mostly stated as the R-factor in the universal soil loss equation (USLE) and its revised version, RUSLE (Panagos et al. 2015). Due to scarcity of data to estimate the R-factor, this study estimates the rainfall erosivity in the investigated area using rainfall data.

Oduro-Afriyie (1995) used the Fournier (1960) index (FI), defined as:

1 $$\frac{{P_{max}^{2} }}{P}.$$

$p_{max}$, the rainfall amount in the wettest month and P is the annual rainfall amount, to compute rainfall erosivity indices for various stations in Ghana. This FI index has limitations as an estimator of the rain erosivity index because low amounts of monthly rainfall can have erosive power. It is irrational that if the maximum monthly rainfall $p_{max}$ remains the same with an increase mean annual rainfall, the (FI) decreases since an increase in total rainfall should result in an increase of erosivity (Deyanira and Donald 2005).

Based on these limitations, Arnoldus (1980) modified the (FI) index into a modified Fournier index (MFI) including the amount of rainfall of all the months in the year:

2 $$MFI = \mathop \sum \limits_{i = 1}^{12} \frac{{p_{i}^{2} }}{P}.$$

with *p*_*i*_ the monthly rainfall amount for the ith month (mm) and P: the annual rainfall amount (mm), Lujan and Gabriels (2005). This study, therefore, estimates the rainfall erosivity of the GAEC site using the Modified Fournier Index, Arnoldus (1980).

## Study area

**Fig. 1:**
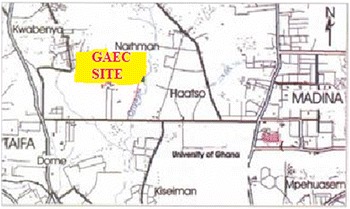
Location map of GAEC

The GAEC is the state institution involved in the peaceful uses of radiation a surveillance of the utilization of nuclear and radioactive sources in Ghana. GAEC is located north-west of the University of Ghana. It is about 24 km from the central Accra and 6 km off the Legon–Madina road towards Kwabenya through the Haatso Township. The area lies within latitudes 5°6′7’’N to 5°6′9’’N and longitudes 0°21′W to 0°26′W at elevation of 64 m (Fig. 1).

**Fig. 2:**
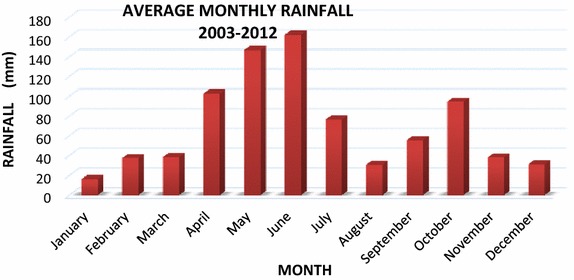
Mean monthly rainfall Source of data: Ghana Meteorological Services Dept. Accra

The climate of the site is equatorial with two rainy and two dry seasons (Fig. 2). There is a dry season from November to March during which rainfall is around 32 mm per month. This season is followed by a rainy season from April to June during which an average of about 125 mm rain falls per month. There is a slight dry season from July to August after which there is another rainy season. The mean annual rainfall is 830 mm.

The highest mean monthly temperature of about 30 °C occurs between March and April and the lowest of about 26 °C in August. The highest mean monthly relative humidity does not exceed 75 %, and the lowest is about 60 % (Dickson and Benneh 2004).

The broad valley of the Onyasia river flanks the site on its eastern margin, swampy conditions are generally found in the north-east of the site. During the wet season, small localised swamps develop which may persist well into the dry season. Surface run-off in this area is very low as the top-soil is everywhere sandy. However, after heavy storms there may be some movement of water over the clay horizon below the sandy top-soil (Akaho et al. 2003).

## Methods

Monthly rainfall data for the period 2003–2012 for Kwabenya area was obtained from the Ghana Meteorological Services Department, Accra, Ghana. The mean monthly and annual rainfall values for various years were computed and the corresponding rainfall erosivity indices for those years for the area were computed using the MFI. The mean rainfall erosivity index for the site was then calculated.

The degree of relationship between the annual rainfall and the annual rainfall erosivity was established using the Pearson’s Coefficient of Correlation given by;

3 $$r = \frac{\sum XY}{{N\sigma_{x} \sigma_{y} }}$$

where r = product moment correlation coefficient; $x = (X - \bar{X})$, $y = (Y - \bar{Y})$; *σ*_*x*_ = Standard deviation of series X; *σ*_*y*_ = Standard deviation of series Y; N = Number of pairs of observation.

## Results and discussion

**Fig. 3:**
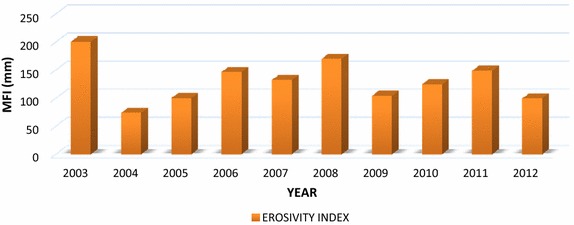
Annual rainfall modified fournier index (MFI) for GAEC SITE

The results of the annual rainfall erosivity estimated for the GAEC site (2003–2012) through the adoption of the MFI is presented in Fig. 3. The mean annual rainfall erosivity was 129.8 mm at the site. This fell within the high erosivity category, as can be interpreted from Table 1. From Fig. 3, the highest rainfall erosivity index of 200.4 mm depicting very high erosion risk, was recorded in 2003 while the lowest, 73.5 mm depicting low erosion risk, was recorded in 2004.

**Table 1:** Rainfall erosivity index classification based on the modified Fournier index (MFI) (Balogun et al. 2012)

MFI range (mm)	Interpretation (erosion risk class)
<60	Very low
60–90	Low
90–120	Moderate
120–160	High
>160	Very high

## Annual rainfall: annual rainfall erosivity index relationship

**Fig. 4:**
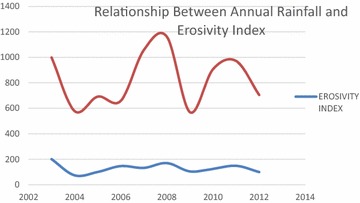
Relationship between annual rainfall and erosivity index

The calculated Pearson’s correlation coefficient r value of 0.7 (at 0.01 level of significance) showed a positive degree of relationship between the amount of annual rainfall and the corresponding rainfall erosivity index (Fig. 4).

## Analysis of annual rainfall erosivity

Yearly variation of the erosivity index shows an intermittent trend as depicted in Table 2. The mean annual erosivity index (129.8 mm) revealed that the site is in the high erosion risk zone. The estimated high erosivity index for the study area presages further risk of soil erosion hazards, especially under conditions of increasing rainfall. There is therefore the need to develop soil protection and management strategies to protect the soil from erosion.

**Table 2:** Yearly variation of Fournier erosivity index for the study area

Year	Erosivity index	Erosion class
2003	200.4	Very high
2004	73.5	Low
2005	99.9	Moderate
2006	146.3	High
2007	132.0	High
2008	169.9	Very high
2009	104.2	Moderate
2010	124.2	High
2011	148.6	High
2012	99.4	Moderate
